# The views and perceptions of water immersion for labor and birth from women who had birthed in Australia but had not used the option

**DOI:** 10.18332/ejm/150355

**Published:** 2022-08-04

**Authors:** Megan Cooper, Jane Warland

**Affiliations:** 1 College of Nursing and Health Sciences, Flinders University, Adelaide, Australia; 2 Curtin School of Nursing, Faculty of Health Sciences, Curtin University, Perth, Australia

**Keywords:** childbirth, choice, experience, water immersion, waterbirth

## Abstract

**INTRODUCTION:**

Recent research highlights that women experience great benefits from immersing in warm water during labor and birth. While there has been an increase in research examining women’s experiences of using water, there has been little investigation of the views and perceptions of women who have not. The objective of this study was to examine the views and perceptions of water immersion from women who had birthed in Australia but had not used the option.

**METHODS:**

An e-survey was distributed to women using purposive and snowball sampling methods between November 2016 and October 2017. Email, text, social media, and parenting forums maximized recruitment. A total of 395 women who had not used water immersion for labor or birth participated.

**RESULTS:**

Three quarters of all women surveyed suggested that they would have considered using the option of water immersion if it was offered to them. Nearly 20% of all women did not know it was an option and, therefore, were only made aware of it as a result of completing this survey. Women indicated that they most often learned about water immersion from a midwife. When asked to rate the benefits and concerns, the majority held very little concern and generally agreed that water immersion would probably provide the associated benefits that are commonly cited in the literature.

**CONCLUSIONS:**

Water immersion offers women many benefits although may not always be discussed antenatally. In light of these results, water immersion could be included in the discussions about labor and birth options antenatally and better supported during labor and birth.

## INTRODUCTION

Choice surrounding childbearing options is important to women. Choice, when facilitated and supported, is not only a key tenet of maternity care provision but it is also influential in how women perceive and experience their childbearing experience. Decision making and more, autonomous decision making, is pivotal in increasing women’s sense of control^1,2^. When women have a sense of control, they are subsequently more likely to experience childbearing as a positive experience and, therefore, more likely to report higher levels of satisfaction^1-3^. Conversely, women who experience a loss of control and who are hampered in their ability to make informed decisions, experience an increased risk of psychological trauma and adverse pregnancy outcomes^4^.

Standard one of the Australian Midwifery Practice Standards states that a midwife ‘identifies what is important to women as the foundation for using evidence to promote informed decision-making, participation in care, and self-determination’ while standard two states that the midwife ‘supports the choices of the woman, with respect for families and communities in relation to maternity care’^5^. These aspects of care are essential in facilitating partnership and the midwifery philosophy of woman-centered care through which the partnership model is fostered^6-8^. This partnership is strengthened through the provision of evidence-based information which ultimately encourages women to seek the options that are most relevant to their individual circumstances across pregnancy, birth, and their transition to parenthood^9^.

In Australia, policies and guidelines pertaining to the use water immersion for labor and birth have been largely restrictive, inhibiting women’s ability to access the option even where they are eligible^10,11^. In a study by Cooper et al.^12^, it was found that water immersion was often not advertised as a viable option. Women had to seek water immersion rather than it being offered as part of routine discussions about labor and birth. Some policies and guidelines explicitly stated that water immersion was not to be promoted or encouraged^13^. This was further compounded by the inaccessibility to an appropriately trained clinician and/or suitable bath or pool^14^. Despite this, policies and guidelines were found to be a facilitative mechanism in some circumstances. That is, where they were written to reflect the current evidence base, these documents supported women in their self-determination and choice surrounding water for labor and birth^15^.

Newnham et al.^11^ highlighted that water immersion was often portrayed as an option associated with risk. On comparing the information provided to women about water immersion with similar resources for epidural, they found that epidural was portrayed positively. Risks associated with the epidural were accepted as a ‘medically tolerable’ whereas risks associated with water birth were portrayed as ‘tolerable to women’^11^. This was also discussed by Bryers and Van Teijlingen^16^:

*‘When a woman who has had a previous caesarean section chooses to have a waterbirth, the midwife is put in a difficult position: she may wish to support the woman, but to do so will mean that she … is practicing outside the agreed clinical guidelines. Both the midwife and the woman will face considerable pressure from the dominant obstetric ideology; that this is not safe. However, the medical approach to this case is likely to see an epidural in labor as an acceptable risk because this is perceived as a technology which can be controlled by continuous monitoring; it is arguable whether this is 'an optimum level of care' but this is how it will be perceived as it is supported by the authoritative knowledge.’*

They argued that this was reflective of entrenched views about birth and the influence that the biomedical system has over women’s bodily autonomy and freedom to exercise choice.

There is evidence to suggest that women are not always made aware of all the options available to them and, even where they are, they are not always able to actively exercise choice^11,12^. With regard to water immersion for labor and birth, women’s choice has often been further hindered by limited evidence defining and quantifying risk to both the woman and her baby. As such, questions of risk related to warm water immersion still ensue. The most common concerns relate to the baby inhaling or aspirating water, cord avulsion, perineal trauma, and estimation of blood loss, maternal collapse, and the associated challenge of evacuating women from a pool/bath where resuscitation is required^15,17,18^. In contrast, many studies have concluded that water immersion is associated with less intervention, little or no difference in rates of spontaneous vaginal birth and no increased adverse events for the woman or baby during the first or second stages of labor^17,18^. Further to this, a recent national cohort study of 6264 waterbirths in the UK concluded that there was no association between waterbirth and low Apgar scores but there was an association between waterbirth and reduced incidence of admission to neonatal units and postpartum hemorrhage (PPH)^18^. While the latest Cochrane review concludes that there appears to be no evidence of harm, there is a call for more research^17^.

From a psychological perspective, water immersion appears to be aligned with a more positive birth experience, offering women much more than just pain relief^15,17-20^. For example, a recent meta-thematic synthesis of women’s experiences of water immersion for labor and birth, highlighted that the option promotes a sense of agency and supports the woman as a complete person^19^ while a study of more than 700 women highlighted that women rate water immersion highly against commonly cited benefits^20^.

However, what is not known, is the extent and level to which women are made aware of the option of water immersion during their pregnancy. There is also no study that has explored the views and perceptions of water immersion from women who have not used the option. In recognizing this gap in the literature, this study surveyed women who had not used water immersion during labor and birth and asked them to share insight into the antenatal education they received during their most recent pregnancy. Their views and perceptions of the commonly cited benefits and risks related to water immersion were also explored.

## METHODS

This study used an e-survey to explore the views and perceptions of water immersion for labor and birth from women who had not used the option.

### Survey development

A survey based on existing literature was developed. It focused on the information women had received during their antenatal care with respect to water immersion and other pain relief options. Women’s views and perceptions of the commonly cited benefits and risks surrounding water immersion were also sought. The survey included three sections. Section one asked participants to answer a range of demographic related questions; section two asked questions about the information received through antenatal care related to water immersion and other pain relief options; section three asked participants to rate their perceptions and views of the benefit and risks related to water immersion on Likert scales (adapted from previous studies by the authors). The survey questions were reviewed by an expert panel of midwives and four consumers for face and content validity. Questions were adjusted based on feedback received from this review process.

The survey was hosted on the SurveyMonkeyTM platform. Question types included dichotomous, multiple choice and Likert scales, and branching logic was used where necessary. Once entered into the SurveyMonkeyTM platform, four consumers (who had recently given birth and used water immersion) tested the survey to ensure that it was functioning correctly and that branching questions navigated to the correct page. Changes were made in response to feedback received from these four consumers prior to the survey going live. These four consumers were excluded from participating in the study.

### Context

The primary study was conducted in Australia. Maternity care provision in Australia is largely offered through public and private hospitals. Of the more than 0.3 million births each year, 97% occur in hospital and less than 1% occur in the woman’s home^21^; 45% of women will have a midwife employed by the public sector as their designated lead maternity care provider^22^. While most women will be cared for by a midwife at some time during their childbearing experience, many women receive principal care from a private obstetrician (11.5%); some irrespective of their risk status. Latest data suggest that 30% of women receive continuity of care by a known care provider for the whole maternity period^22^. Despite this, maternity care in Australia has often been described as fragmented, with women seeing multiple care providers throughout their pregnancy, birth, and postnatal experience.

### Population and sample

Women from all States and Territories across Australia were eligible to participate if they had birthed in Australia. Women who had birthed outside Australia were not included. A total of 395 women responded to the survey between November 2016 and October 2017. There was no time limit placed on participation to maximize recruitment. This is acknowledged as a weakness of the study due to potential recall bias.

### Data collection

Purposive and snowball sampling methods were used to maximize recruitment. The survey was distributed via email, text, and social media. Facebook and Twitter proved to be useful recruitment strategies. Administrators of childbirth and parenting forums were also contacted. Where approval was gained, a dedicated post with the survey link was added to a relevant forum. Potential participants were informed that the study aims were: 1) to explore the information offered about water immersion for labor and birth by clinicians during their antenatal care; and 2) to seek their views and perceptions of water immersion.

Participants accessed the survey via a hyperlink. The survey took participants between 10–15 minutes to complete. Answers to questions were able to be changed prior to the participant completing the survey and access was restricted by IP address to ensure that participants were in Australia and not able to complete the survey more than once. Participants were able to return to the survey via a password if they wished. Participants were not provided with an incentive to complete the survey.

### Rigor

The final survey used in this study was screened for face and content validity by a panel of experienced professionals and four consumers who had used water immersion. These consumers were excluded from participating in the study. Questions including the Likert scales included in section three of the survey were adapted from previous studies related to water immersion. The original Likert scales had previously been tested for validity and reliability through test-retest^15,23^. Minor wording and language changes were made.

### Ethical considerations

The Human Research Ethics Committee (HREC) of the University of South Australia approved the study. All data were collected anonymously. While demographic data such as age and education level were collected, any information that identified the participant was not collected. An electronic participant information sheet was included at the beginning of the survey and consent was implied if participants completed the survey. Questions asked were not compulsory to ensure that participants had discretion over what they wished to answer. During analysis, no one individual participant could be identified due to pooling of the data.

### Data analysis

Survey results were collected and collated via SurveyMonkeyTM and using the inbuilt functions of the platform, trends were explored prior to the full data set being downloaded to Statistical Package for the Social Sciences (SPSS) version 25. Data were predominantly analyzed descriptively by percentage and mean.

## RESULTS

### Participant demographics

Most participants were aged 20–39 years (n=345/395; 87.3%), held a degree (n=142/395; 35.9%) and identified as Australian (n=345/395; 87.3%). Most women resided in South Australia (n=142/395; 35.9%). A total of 67 women (17.0%) were pregnant at the time of completing the survey. For full demographic data see Table 1.

**Table 1:** Demographic characteristics of the sample, November 2016 to October 2017, Australia (N=395)

*Characteristics*	*n (%)*
**Age** (years)	
<20	3 (0.8)
20–29	134 (33.9)
30–39	211 (53.4)
40–49	45 (11.4)
Prefer not to answer	2 (0.5)
**Education level**	
Primary school	1 (0.3)
Secondary school	51 (12.9)
Diploma/certificate	107 (27.1)
Degree	142 (35.9)
Master’s/Honors degree	74 (18.7)
PhD	8 (2.0)
Other	12 (3.0)
**Ethnic background/nationality**	
Australian	354 (87.3)
Asian	5 (1.3)
British	17 (4.3)
Middle-Eastern	2 (0.5)
European	19 (4.8)
African	3 (0.8)
American	1 (0.3)
Other	3 (0.8)
**Residence**	
New South Wales	61 (15.4)
Victoria	74 (19.0)
South Australia	142 (35.9)
Tasmania	3 (0.8)
Western Australia	29 (7.3)
Queensland	70 (17.7)
Australian Capital Territory	8 (2.0)
Northern Territory	7 (1.8)
**Current relationship status**	
Married	290 (73.4)
Widowed	1 (0.3)
Divorced	5 (1.3)
Separated	11 (2.8)
In a domestic partnership or civil union	70 (17.7)
Single, but cohabiting with a significant other	5 (1.3)
Single, never married	13 (3.3)
**Number of children**	
1	166 (42.0)
2	145 (36.7)
3	53 (13.4)
4	20 (5.1)
≤5	11 (2.8)
**Pregnant**	
Yes	67 (17.0)
No	328 (83.0)
**Place of birth**	
Public hospital	268 (67.8)
Private hospital	84 (21.3)
Birth center	13 (3.3)
Home	23 (5.8)
Other	7 (1.8)

### Information about water immersion for labor and birth

All women who completed the survey were asked to reflect on their most recent experience of pregnancy and childbirth and the information that was provided to them about water immersion for labor and birth. Of the 395 women who responded, 313 (79.2%) were aware that water immersion was an option while 76 (19.2%) of these women did not know that it was an option. When exploring whether or not these participants would use water immersion if it was offered to them, 62 responded; 50% (n=31) answered ‘yes’, 29% (n=18) were not sure, 11% (n=7) answered ‘yes, but I didn’t meet the criteria’ and the remaining 10% (n=6) answered ‘no, it doesn’t interest me’. A total of 260 (65.8%) stated they were not provided with information about water immersion during their pregnancy while 129 (32.7%) suggested that they received information, however 69 (17.5%) suggested that they would have liked more information.

Of those who were aware that water immersion was an option and answered the question: ‘Who provided you with information and/or made you aware of the option of water immersion for labor and/or birth?’, 141 (n=284; 45.1%) were informed by a midwife, 74 (n=284, 23.6%) read about it on the internet and 27 (n=284; 8.6%) heard about it from a friend. Seven (n=284; 2.2%) were made aware of the option by obstetricians. Other (n=28/284; 9.0%) responses included antenatal classes, word of mouth, books, magazines, doula and ‘I am a midwife.’

When asked if the person/source portrayed the option of water immersion as safe, most participants indicated that the use of water for both labor and birth was discussed as being safe (n=203/284; 71.5%) and 59 (20.8%) suggested that it was only discussed as safe for labor. The remainder suggested that the person/source portrayed water immersion as unsafe (n=22/284; 7.7%). Almost two-thirds (n=181/284; 63.7%) of the women suggested that the person/source outlined the benefits of water immersion while only one-third (n=95/284; 33.5%) suggested that risks were discussed. For full results see Table 2.

**Table 2:** Information provided to women about water immersion during a previous pregnancy, November 2016 to October 2017, Australia (n=395)

*Questions/Answers*	*n (%)*
**Did you receive information about the option of water immersion?**	
Yes, information was thorough	60 (15.2)
Yes, but I would have liked more information	69 (17.5)
No, but I was aware that it was an option	184 (46.6)
No, I didn’t even know it was an option	76 (19.2)
Missing	6 (1.5)
Who provided you with information and/or made you aware of the option of water immersion for labor and/or birth?*	
Midwife	141 (45.1)
Obstetrician	7 (2.2)
General Practitioner (GP)	0 (0)
Family member	7 (2.2)
Friend	27 (8.6)
I read about it on the internet	74 (23.6)
Other	28 (9.0)
Missing*	29 (9.3)
In your opinion, did this person/source portray water immersion as a safe option?*	
Yes, for both labor and birth	203 (71.5)
Yes, but only for labor	59 (20.8)
No	22 (7.7)
Did this person/source discuss any risks related to water immersion for labor and/or birth?*	
Yes	95 (33.5)
No	101 (35.6)
I can’t recall	88 (30.9)
Did this person/source discuss any benefits related to water immersion for labor and/or birth?*	
Yes	181 (63.7)
No	56 (19.7)
I can’t recall	47 (16.6)

* n=284.

### Pain relief options discussed

Participants indicated that the most common pain relief options discussed during the most recent pregnancy were gas/nitrous oxide (n=274/395; 69.4%), epidural (n=263/395; 66.6%) and the shower (n=223/395; 56.5%). The options discussed least were fentanyl (n=26/395; 6.6%) and sterile water injections (n=12/395; 3.0%). Nearly 10% suggested that pain relief options were not discussed. Other options included ‘panadeine forte’ and ‘I knew what I wanted’ as examples. Full results are presented in Table 3.

**Table 3:** Pain relief options discussed in a prior pregnancy, November 2016 to October 2017, Australia (N=395)

*Pain relief option*	*n (%)*
Gas/nitrous oxide	274 (69.4)
Epidural	263 (66.6)
Shower	223 (56.5)
Heat pack	171 (43.3)
Massage	168 (42.5)
TENS machine	142 (35.9)
Pethidine	137 (34.7)
Hypnobirthing/meditation	85 (21.5)
Morphine	82 (20.8)
Spinal anesthesia	60 (15.2)
Acupuncture/acupressure	34 (8.6)
General anesthesia	29 (7.3)
Fentanyl (injection/nasal spray)	26 (6.6)
Sterile water injections	12 (3.0)
Pain relief options not discussed	39 (9.9)
Other	19 (4.8)

### If you had the opportunity to use water immersion, would you use it?

Women who responded to the survey were asked if they would use water immersion if they had the opportunity; 75.2% (n=297/395) of all women answered ‘yes’, however 66 (16.7%) of these women suggested that they had been told that they did not meet the criteria. Another 41 (10.4%) women suggested that they were not interested in using water and 57 (14.4%) were not sure.

Of those women who indicated that they did not meet the criteria for using water, the main reasons selected against a range of options were: ‘my body mass index (BMI)/weight was too high’ (n=23/66; 34.8%), ‘I was induced’ (n=21/66; 31.8%), and ‘I had a previous cesarean section’ (n=16/66; 24.2%). Of the women, 14 (21.2%) suggested that the option was not available, 11 (16.7%) suggested that necessary equipment was not available while 6 (9.1%) suggested that there was not an experienced water immersion practitioner available. Other responses included twin pregnancy, previous hemorrhage, preterm in a previous birth, and Group B Streptococcus. One stated: ‘was told yes water birth and went into labor and was told no’. Women could select more than one response to this question. For all the reasons, see Table 4.

**Table 4:** Reasons why water immersion was not available among women who had been told that they did not meet the criteria, November 2016 to October 2017, Australia (N=66)

*Reasons*	*n (%)*
The hospital didn’t offer the option	14 (21.2)
An experienced water immersion practitioner was not available	6 (9.1)
Necessary equipment was not available (i.e. pool, bath, hot water)	11 (16.7)
The bath/pool was not filled in time	3 (4.6)
My body mass index (BMI)/weight was too high	23 (34.8)
I lost a lot of blood in my previous birth (postpartum hemorrhage)	5 (7.6)
I was told that my baby was too big	6 (9.1)
I was told that my baby was too small	3 (4.6)
My last baby’s shoulders got stuck (shoulder dystocia)	1 (1.5)
I went into labor too early (I had a preterm birth)	3 (4.6)
I went overdue or past my due date	5 (7.6)
I was induced	21 (31.8)
I had a previous cesarean section	16 (24.2)
I needed to have a cesarean section	11 (16.7)
My baby was in distress (my baby’s heart rate was too high or too low)	6 (9.1)
I had high blood pressure (e.g. pre-eclampsia, pregnancy-induced hypertension, pre-existing hypertension)	5 (7.6)
The practitioner supporting me did not agree with water immersion	4 (6.1)
No one told me it was an option	4 (6.1)
I had gestational diabetes	7 (10.6)
I had type 1 or type 2 diabetes	0 (0.00)
Other	10 (15.2)

### Perceived benefits and concerns related to water immersion

**Figure 1:**
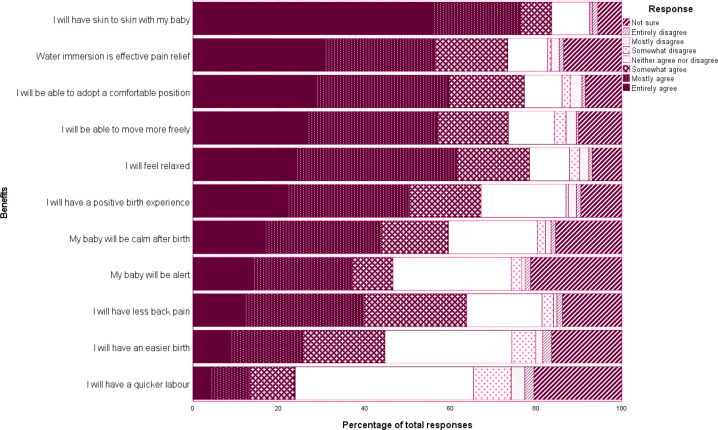
Perceived benefits of water immersion by percentage of responses, November 2016 to October 2017, Australia (n=395)

All participants were asked: ‘to what extent do you agree or disagree with the following statements relating to the use of water immersion for labor and/or birth based on what you know or what you have been told’ for commonly cited benefits and risks. Participants indicated that they agreed with the statements: ‘I will have skin-to-skin with my baby’ (mean 6.24), ‘water immersion is an effective pain relief’, ‘I will be able to adopt a comfortable position’ and ‘I will be able to move freely’. They least agreed (as reflected by the response entirely disagree) with the statements: ‘I will have a quicker labor’ and ‘I will have an easier birth’. Distribution of responses given in Figure 1.

**Figure 2:**
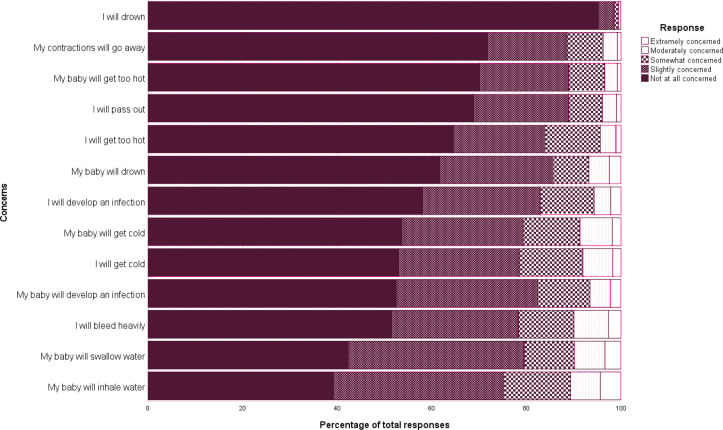
Perceived concerns of water immersion by percentage of responses, November 2016 to October 2017, Australia (n=395)

Participants, overall, did not indicate a high level of concern against the presented statements. The most concern was held for the baby inhaling or swallowing water. They had the least concern for themselves drowning or their contractions going away. Distribution of results are presented in Figure 2.

## DISCUSSION

This study examined the views and perceptions of women who had not previously used water immersion for labor and birth. While a recent study compared the experiences of women who did and did not achieve a water birth^24^, this appears to be the first study to specifically target women who did not or had not planned to use water immersion or were not aware of the option. The results suggest that for this cohort water immersion for labor and birth is not always readily discussed or promoted. While this may be due to the clinician determining that the woman is ineligible for water immersion, these results suggest that over 40% of those who were unaware of the option, would have considered using it if was offered to them. This aligns with previous research that suggests that women must actively seek the option rather than wait for it to be offered^15,25^. They also rated the benefits of water immersion relatively high, and concerns relatively low against all Likert scales.

Participants indicated that the pharmacological methods of epidural and gas/nitrous were the most discussed pain relief options during their antenatal care, which aligns with the findings of Newnham et al.^11^. In addition to this, more than half of the women surveyed suggested that the shower had been discussed. This could suggest that practitioners are aware of the benefits that warm water offers as a pain-relieving measure but are less inclined to support the option of warm water immersion. This could be attributed to a negative view of water immersion, the service not facilitating the option, a lack of available staff or infrastructure, or the logistical challenge of getting women out of a bath/pool if they collapse; all of which have been identified as barriers to water immersion in previous studies^12,15^. Women in this study identified these factors as reasons why they were not able to use water immersion during their most recent birthing experience.

The finding that women were not always made aware of or offered the option supports anecdotal evidence that midwives and other healthcare providers are actively discouraged from offering the option of water immersion. This is now supported by research^25,26^. In Australia, women generally must actively seek out and request the option. Whether or not they are able or eligible for water immersion is further influenced by logistical, staffing, and infrastructural barriers. For example, women are often precluded from using water due to a lack of appropriately trained staff. These factors appear to be pivotal in the decisions around the woman’s intended place of birth. That is, women will seek out the option of water immersion and, where the institution is not able to facilitate the option, they may choose to birth at home with or without the support of a healthcare provider^27^. While the number of women making this choice is low, it does beg the question as to why mainstream maternity care is not always supportive of options that women value, particularly given that these options are associated with positive outcomes^28^.

These positive outcomes also extend to the experiences of women who are, by policies and guidelines, precluded from using the option of water immersion. Women who were interviewed in a study by McKenna and Symon^29^ and actively sought a water birth after a previous cesarean, suggested that they felt empowered, more in control of their birthing experience and experienced both physical and psychological benefits. Further to this, Townsend et al.^30^ found that women who achieved a waterbirth while undergoing a vaginal birth after cesarean referred to the experience as ‘life changing’. In contrast, this study reflects that a previous cesarean section was identified as a reason why women were not able to access the option.

The most common reasons for women not being able to access water immersion in this study were a high body mass index (BMI) and induction of labor. Previous research has attributed this to the possibility of maternal collapse and the associated difficulties of evacuating the woman from the bath or pool^12,31^. While commonly listed as contraindications for water use during labor and birth, there is very little research to support exclusions on the basis of maternal collapse and body mass index as examples^12,31^. In fact, Swann and Davies^32^ and Benko^33^ argue that water immersion may benefit women with high BMI given the noted benefits of ease of movement and position change. With regard to induction of labor, it could be argued that water immersion is a legitimate option, especially now that waterproof telemetry is available and allows for monitoring of the baby during labor and birth.

Women should have the ability to exercise choice and autonomy with respect to childbearing, particularly where a woman’s decision making appears to be limited to those options that are viewed as safe and appropriate^10-13,15^. Water immersion as an option, seems to fall outside of what is deemed safe and necessary^17,28^. This may explain why nearly 70% of participants were not provided with information about the option during their most recent pregnancy. Possible rationale for this could include clinicians deeming the woman ineligible. Despite this, 75% of women surveyed indicated that they would consider using water immersion if it was offered and available to them.

Autonomy and self-determination are recognized as significant contributors to the way in which women view their birth experience in terms of overall satisfaction^2,19^. Autonomy and self-determination contribute to a ‘sense of agency’ and the level of perceived control women have across pregnancy, birth and into the postnatal period^2,19,34^. It is these very factors that underpin the midwifery philosophy of woman-centered care^7,8^. With ever-growing evidence to suggest that water immersion supports not only these factors but the ability of a midwife to support the woman-centered philosophy, it is time that water immersion is recognized and routinely offered as a legitimate option.

### Implications for research and practice

While there is increasing investigation of women’s experiences of water immersion, this is the first study to explore the views and perceptions of those who have not used the option. It is hoped that this study will promote other studies that seek to understand the experiences of all women accessing maternity care, especially those who may not have had the chance to use water immersion. Specifically, further exploration of the barriers women face in accessing water immersion and the subsequent choices they make regarding place of birth, need further investigation.

Understanding women’s experiences of maternity care is key to improving women’s satisfaction, especially given that their experiences of labor and birth can have a direct bearing on their psychological and mental wellbeing. This study challenges clinicians to consider strategies to improve antenatal discussions surrounding options for labor and birth, so that women are able to make informed decisions. It may also prompt clinicians to consider how choice and autonomy related to water immersion can be facilitated.

### Strengths and limitations

A total of 395 women were surveyed. We acknowledge that it is likely that participants held strong views about water immersion and therefore were more likely to participate. Despite this, we identified that nearly one-fifth of the participants were not aware of the option.

While a survey provides a snapshot of women’s views, we acknowledge that this is still a small number of participants compared to the number of births that occur in Australia every year. We also acknowledge that we have not supported these findings with women’s stories; this is planned and will provide further insight into their experiences.

A major limitation of this study is the high likelihood of recall bias given that there was no restriction on the time elapsed since the woman had given birth. We acknowledge this may have influenced the reliability of the results, but this was a strategic move to overcome the challenges of engaging women in a survey that asked them about an experience that they did not have. We attempted to mitigate against this by including women who were also pregnant at the time of survey completion.

We have chosen to report only descriptive statistics in this study, given the sample size. A larger sample size would have allowed further analysis to determine trends and associations based on demographic data and other influencing factors. We acknowledge that this is a limitation.

The results are also not able to be generalized to other populations, particularly given the differences between Australia and other countries. We also were limited in our ability to explore ethnicity with respect to water immersion. However, our results do provide a basis for further research surrounding women’s experiences of receiving information surrounding labor and birth choices.

## CONCLUSIONS

This is the first study to examine the experiences of women who were unaware or did not intend to birth in water and as such, it addresses a current gap in the literature. The findings align with growing evidence that suggests that women are often limited in their ability to exercise choice and autonomy, despite these factors being central to the midwifery philosophy of woman-centered care. Women in this study indicated that they may have considered the option of water immersion if they were aware of it, and it had been discussed with them. This suggests that there is a need for practitioners to discuss this option with woman and advocate for options that women value.

## Data Availability

The data supporting this research are available from the authors on reasonable request.
